# Supplementary material for: Dental findings frequently overlooked in sinus computed tomography reports
Source: PLoS One. 2024 Apr 30;19(4):e0299489. doi: 10.1371/journal.pone.0299489 (PMC11060568; doi:10.1371/journal.pone.0299489)
Supplement: Attachment — Submitted filename: COMMENTS.docx [file pone.0299489.s001.docx]

**Manuscript reviewed: Dental findings frequently overlooked in sinus computed tomography reports**

Type: Research Article
Ref: Submission: PONE-D-23-33980
Reviewer name: Dr Sushma Bommanavar
Brief Summary of the Manuscript reviewed: the original study is an exploratory study aimed to determine how maxillary dentition is initially interpreted in rhinosinusitis patients’ CT/CBCT reports and which findings in particular are potentially missed.

Overall impression of the work, noting both strengths and weaknesses: The strength lies in exploratory hypothesis of finding out the potentially missed area in interpretation of CT/CBCT reports among rhinosinusitis patients. The weakness lies in consideration of only one pathology for interpretation. However, this can be quiet minimal weakness as the study highlights the strength part more as diagnostic accuracy studies that can help in future clinical practices.

Introduction: Well Written

Methodology: Well Written

Result: Well Written

Discussion: Well Written

The original article can be considered if the following revisions are done:

**MAJOR ISSUES:**
1. Kindly follow the AGREE checklist (BMJ 2016;352:i1152 ) & STRAD checklist, **if applicable**

**MINOR ISSUES:**    
1. In figure legend description for figure no 2 & 3, kindly reduce the total word count.
