# Supplementary material for: Dental findings frequently overlooked in sinus computed tomography reports
Source: PLoS One. 2024 Apr 30;19(4):e0299489. doi: 10.1371/journal.pone.0299489 (PMC11060568; doi:10.1371/journal.pone.0299489)
Supplement: Attachment — Submitted filename: Response to Reviewers.docx [file pone.0299489.s002.docx]

Dear Dr Mohmed Isaqali Karobari,

Thank you for your letter and for the reviewers’ encouraging and valuable comments on our manuscript entitled “Dental findings frequently overlooked in sinus computed tomography reports”. All of these comments were very helpful for revising and improving our paper. We have studied these comments carefully and have made corresponding corrections that we hope will meet with your approval. The changes in the revised manuscript are marked by Track changes. In addition, we provide a point-by-point response to the reviewers’ comments below. We hope that the manuscript is now acceptable for publication in its present form. If you have any further queries, please do not hesitate to contact us.

Kind regards,

Annina Wuokko-Landén DDS

annina.wuokko@fimnet.fi

**Journal Requirements:**

1. Please ensure that your manuscript meets PLOS ONE's style requirements, including those for file naming. The PLOS ONE style templates can be found at 
https://journals.plos.org/plosone/s/file?id=wjVg/PLOSOne_formatting_sample_main_body.pdf and 
https://journals.plos.org/plosone/s/file?id=ba62/PLOSOne_formatting_sample_title_authors_affiliations.pdf

**1. Response:**

We thank you for providing the additional information. We have checked the manuscript to ensure that it adheres to the style requirements, and have made some adjustments to the text headings (bolding and italics) as necessary.

2. We note that the grant information you provided in the ‘Funding Information’ and ‘Financial Disclosure’ sections do not match. When you resubmit, please ensure that you provide the correct grant numbers for the awards you received for your study in the ‘Funding Information’ section.

**2. Response:**

We have now included the grant number (grant number is Y1014SU011/1149010/30101) to the Disclosure statement as requested (see pg 1).

3. Thank you for stating the following financial disclosure: 
"This study was supported by the Helsinki University Hospital Research Fund (statistical analysis)."

Please state what role the funders took in the study.  If the funders had no role, please state: "The funders had no role in study design, data collection and analysis, decision to publish, or preparation of the manuscript." 
If this statement is not correct you must amend it as needed. 
Please include this amended Role of Funder statement in your cover letter; we will change the online submission form on your behalf.

**3. Response:**

The cover letter and title page have been revised to include the following statement (see pg 1):

‘The funders had no role in the study design, data collection and analysis, decision to publish, or the preparation of the manuscript.’ The funding disclosure statement was changed to ‘Statistical analysis of this study was financially supported by the Helsinki University Hospital Research Fund (grant no. Y1014SU011/1149010/30101).’

4. We note that your Data Availability Statement is currently as follows: [All relevant data are within the manuscript and its Supporting Information files.]

Please confirm at this time whether or not your submission contains all raw data required to replicate the results of your study. Authors must share the “minimal data set” for their submission. PLOS defines the minimal data set to consist of the data required to replicate all study findings reported in the article, as well as related metadata and methods (https://journals.plos.org/plosone/s/data-availability#loc-minimal-data-set-definition).

For example, authors should submit the following data:

- The values behind the means, standard deviations and other measures reported;
- The values used to build graphs;
- The points extracted from images for analysis.

Authors do not need to submit their entire data set if only a portion of the data was used in the reported study.

If your submission does not contain these data, please either upload them as Supporting Information files or deposit them to a stable, public repository and provide us with the relevant URLs, DOIs, or accession numbers. For a list of recommended repositories, please see https://journals.plos.org/plosone/s/recommended-repositories.

If there are ethical or legal restrictions on sharing a de-identified data set, please explain them in detail (e.g., data contain potentially sensitive information, data are owned by a third-party organization, etc.) and who has imposed them (e.g., an ethics committee). Please also provide contact information for a data access committee, ethics committee, or other institutional body to which data requests may be sent. If data are owned by a third party, please indicate how others may request data access.

**4. Response:**

All relevant data are provided within the manuscript. Due to the sensitive patient information included in our dataset, the raw data cannot be shared via public repositories.

5. We note that Figures 2 and 3 in your submission contain copyrighted images. All PLOS content is published under the Creative Commons Attribution License (CC BY 4.0), which means that the manuscript, images, and Supporting Information files will be freely available online, and any third party is permitted to access, download, copy, distribute, and use these materials in any way, even commercially, with proper attribution. For more information, see our copyright guidelines: http://journals.plos.org/plosone/s/licenses-and-copyright.
 
We require you to either (1) present written permission from the copyright holder to publish these figures specifically under the CC BY 4.0 license, or (2) remove the figures from your submission:
 
a. You may seek permission from the original copyright holder of Figures 2 and 3 to publish the content specifically under the CC BY 4.0 license. 
We recommend that you contact the original copyright holder with the Content Permission Form (http://journals.plos.org/plosone/s/file?id=7c09/content-permission-form.pdf) and the following text:
“I request permission for the open-access journal PLOS ONE to publish XXX under the Creative Commons Attribution License (CCAL) CC BY 4.0 (http://creativecommons.org/licenses/by/4.0/). Please be aware that this license allows unrestricted use and distribution, even commercially, by third parties. Please reply and provide explicit written permission to publish XXX under a CC BY license and complete the attached form.”
 
Please upload the completed Content Permission Form or other proof of granted permissions as an "Other" file with your submission. 
 
In the figure caption of the copyrighted figure, please include the following text: “Reprinted from [ref] under a CC BY license, with permission from [name of publisher], original copyright [original copyright year].”
 
b. If you are unable to obtain permission from the original copyright holder to publish these figures under the CC BY 4.0 license or if the copyright holder’s requirements are incompatible with the CC BY 4.0 license, please either i) remove the figure or ii) supply a replacement figure that complies with the CC BY 4.0 license. Please check copyright information on all replacement figures and update the figure caption with source information. If applicable, please specify in the figure caption text when a figure is similar but not identical to the original image and is therefore for illustrative purposes only.

**Response:**

All three of the submitted figures were created by our group from our own dataset.

**Response to the Reviewers:**

**Reviewer #1:**

Even though the study examines how maxillary dentition is interpreted in CT/CBCT reports of rhinosinusitis patients, it does not meet rigorous academic standards as it solely relies on observational research and does not establish any statistical correlations. The absence of statistical analyses reduces the study's strength and makes it vulnerable to subjective interpretations. Although the study addresses the significance of dentition information in rhinosinusitis imaging, it lacks a quantitative approach, thereby limiting its ability to contribute to evidence-based knowledge. By incorporating statistical correlations into a more comprehensive methodology, the academic credibility and reliability of the findings would be enhanced.

**Response:**

We thank the reviewer for their careful reading of our manuscript and for their useful and helpful comments on improving our manuscript.

In the Materials and methods section, we have described the statistical methods we employed. The McNemar test was used to compare the originally reported CT/CBCT radiological dental findings with a comprehensive re-evaluation of the images by an oral and maxillofacial radiologist. Kappa coefficients were also calculated to determine the level of agreement between the original reports and the re-evaluation of the scans. We have revised the abstract based on this comment (see pg 2).

**Reviewer #2:**

Dear Authors,

In general, the idea of this study, regarding the investigation of how often and with which precision dental pathology is evaluated in the original CT/CBCT reports of rhinosinusitis patients, whether some findings are overlooked, and how often an insufficient area of interest or artefacts hindered the accurate interpretation of radiological findings concerning teeth is interesting.
The role of these aspects in medicine needs further studies that could open a creative matter of debate in literature by adding new information.
The study was well conducted by the authors; however, there are some concerns to revise that are described below:

- Better formulate the abstract section describing the aim of the study.
- The introduction section resumes the existing knowledge regarding this topic but at the end of this section, Authors should underline the rationale of the study.
- In the central section, Authors should better clarify the inclusion and exclusion criteria.
- Better describe how the sample size of the study was calculated.
- The discussion section appears well organized. Please add a specific sentence that clarifies the results obtained in the first part of the discussion.
- The conclusion should reinforce in light of the discussions.
- Add the strenght of the study.

Best regards.

**Response:**

We thank the reviewer for their careful reading of our manuscript and for their useful and helpful comments on improving our manuscript. We have adjusted the text as follows:

- The Abstract now defines the aim of the study (see pg 2).
- The rationale for this study is now underlined in the Introduction (see pg 3).
- To clarify the inclusion and exclusion criteria, the Materials and methods section has been revised (see pg 4).
- The sample size (n = 300) represents the total number from 759 adult acute/chronic rhinosinusitis patients with a CT/CBCT image and for whom the original report was available. All patients visited our hospital during the same year (see pg 4).
- One sentence is added to the first section of the Discussion section (see pg 10).
- We have slightly modified the Conclusions section (see pp 12–13) and added the strengths of our study to the Discussion section (see pg 12).

**Reviewer #3:**

Manuscript reviewed: Dental findings frequently overlooked in sinus computed tomography reports
Type: Research Article
Ref: Submission: PONE-D-23-33980
Reviewer name: Dr Sushma Bommanavar
Brief Summary of the Manuscript reviewed: the original study is an exploratory study aimed to determine how maxillary dentition is initially interpreted in rhinosinusitis patients’ CT/CBCT reports and which findings in particular are potentially missed.
Overall impression of the work, noting both strengths and weaknesses: The strength lies in exploratory hypothesis of finding out the potentially missed area in interpretation of CT/CBCT reports among rhinosinusitis patients. The weakness lies in consideration of only one pathology for interpretation. However, this can be quiet minimal weakness as the study highlights the strength part more as diagnostic accuracy studies that can help in future clinical practices.
A good study design & well written but needs few modifications to be made.
Introduction: Well Written
Methodology: Well Written
Result: Well Written
Discussion: Well Written

The original article can be considered if the following revisions are done:

MAJOR ISSUES:
1. Kindly follow the AGREE checklist (BMJ 2016;352:i1152 ) & STRAD checklist, if applicable

MINOR ISSUES:
1. In figure legend description for figure no 2 & 3, kindly reduce the total word count.

**Response:**

We thank you for your careful reading of our manuscript and the helpful suggestions for improving it. We have looked into the AGREE checklist and consider it a good tool for future clinical guidelines of odontogenic sinusitis diagnostics and further research. As such, we have added it to the references (see pg 12). As we mentioned in the Discussion section, one limitation of our retrospective study is the inability to verify the possible diagnosis and our inability to categorise pathological dental findings as an obvious potential cause of OS nor as directly causing sinonasal symptoms. Therefore, our study is not precisely a diagnostic accuracy study and, thus, not all STARD checklist points can be fulfilled.

We have condensed the legends for Figures 2 and 3 and reduced the overall word count.
